# Different Modular Organization Between Early Onset and Late Onset Depression: A Study Base on Granger Causality Analysis

**DOI:** 10.3389/fnagi.2021.625175

**Published:** 2021-02-09

**Authors:** Naikeng Mai, Yujie Wu, Xiaomei Zhong, Ben Chen, Min Zhang, Qi Peng, Yuping Ning

**Affiliations:** ^1^Department of Neurology, The Affiliated Brain Hospital of Guangzhou Medical University (Guangzhou Huiai Hospital), Guangdong, China; ^2^School of Psychology, South China Normal University, Guangdong, China; ^3^Department of Geriatrics, The Affiliated Brain Hospital of Guangzhou Medical University (Guangzhou Huiai Hospital), Guangdong, China; ^4^The First School of Clinical Medicine, Southern Medical University, Guangdong, China; ^5^Guangdong Engineering Technology Research Center for Translational Medicine of Mental Disorders, Guangdong, China

**Keywords:** late life depression, Granger causality, brain network, modularity, normalized mutual information

## Abstract

**Background:** Modular organization reflects the activity patterns of our brain. Different disease states may lead to different activity patterns and clinical features. Early onset depression (EOD) and late onset depression (LOD) share the same clinical symptoms, but have different treatment strategies and prognosis. Thus, explored the modular organization of EOD and LOD might help us understand their pathogenesis.

**Method:** The study included 82 patients with late life depression (EOD 40, LOD 42) and 90 healthy controls. We evaluated the memory, executive function and processing speed and performed resting-stage functional MRI for all participants. We constructed a functional network based on Granger causality analysis and carried out modularity, normalized mutual information (NMI), Phi coefficient, within module degree z-score, and participation coefficient analyses for all the participants.

**Result:** The Granger function network analysis suggested that the functional modularity was different among the three groups (*P*_auc_ = 0.0300), and NMI analysis confirmed that the partition of EOD was different from that of LOD (*P*_auc_ = 0.0190). Rh.10d.ROI (polar frontal cortex) and Rh.IPS1.ROI (dorsal stream visual cortex) were shown to be the potential specific nodes in the modular assignment according to the Phi coefficient (*P* = 0.0002, *P*_fdr_ = 0.0744 & *P* = 0.0004, *P*_fdr_ = 0.0744).

**Conclusion:** This study reveal that the functional modularity and partition were different between EOD and LOD in Granger function network. These findings support the hypothesis that different pathological changes might exist in EOD and LOD.

## Introduction

Late life depression (LLD) is a common mental disorder in the elderly, and the morbidity is ~3.6–4.6% (Weyerer et al., 2013). According to differences in the age of onset, LLD can be divided into early onset depression (EOD) and late onset depression (LOD). EOD and LOD share the same clinical symptoms but have different treatment strategies and prognosis. Accumulating evidence has shown that EOD and LOD may have different etiologies. For instance, EOD might be associated with a family history of mental illness (Harald and Gordon, 2012), an anxious personality and stressful events (Miyata et al., 2016). In contrast, LOD might be associated with somatic disease and neurodegenerative disease (Mackin et al., 2014). Different etiologies might lead to different pathological features and different brain activity. However, the evidence supporting differences in the pathology between EOD and LOD is limited. Furthermore, according to previous articles, LOD is likely to be the preclinical stage of Alzheimer's disease (AD) (Dillon et al., 2009; Heser et al., 2016), or to increase the risk of developing AD in the future (Tateno et al., 2015). Therefore, understanding the similarities and differences between EOD and LOD, especially in terms of cognitive function and relative brain activity, is indispensable for understanding the pathogenesis of the disease.

Resting state magnetic resonance(rs-fMRI)is a non-invasive method that helps us to visualize the activity of the brain through the Blood oxygen level dependent (BOLD) signal. Furthermore, rs-fMRI could reflect the fundamental characteristics of individual brain activity. Functional activity was correlated with structural features (Sporns, 2011). Thus, the functional brain activity could indicate the pathological changes between different subjects. Among various analytical methods, brain connectome analysis can provide us with information on brain connectivity and quantify the organization through the definition of nodes and edges.

Functional connectivity is a method of using the BLOD signal to measure the activity consistency between brain regions. It is also a common way to define edges in a functional network. Currently, the main method used to describe functional connections between brain regions uses the Pearson coefficients of the main time series of two regions. However, the activity of the human brain presents time sequence changes even at the resting stage, and the current functional connection based on the Pearson coefficient ignores the time sequence property. Thus, we applied Granger causality (GC) analysis to describe the functional connection between brain regions. GC is a classical statistical method that was first applied in economics to detect the directional influence between different components. This method was recently applied in the analysis of fMRI data, which can help us determine the direction of the network connection and provide a more accurate functional brain network based on the time dependency information process in the human brain in a resting state (Wen et al., 2012a). We believe that the application of GC network analysis can help us understand the functional changes of LLD more comprehensively.

Modularity is a property used to quantify the tendency of the network element to integrate into the same module and the separation between modules (Leicht and Newman, 2008). According to the connectome, modularity has been found in the human brain, and several functional modules were defined (Barnes et al., 2009). A change in the modularity can be found in several neuropsychiatric disorders, such as AD (Brier et al., 2014), Parkinson's disease (Tinaz et al., 2017) and major depressive disorder (Peng et al., 2014), and indicates the pathological changes in these diseases. In LLD, the change in the modularity can also be observed in the functional network, especially in patients with cognitive impairment (Li et al., 2015). A similar result is shown in the analysis of the structural network of LLD (Mak et al., 2016). However, there are contradictory findings for LLD, which failed to find abnormal modularity in LLD (Bohr et al., 2012). Based on the current uncertainty about whether the modularity is changed in LLD and the potential pathological difference between EOD and LOD, we hypothesize that the uncertainty of the findings of modularity in LLD might be caused by differences in the modularity in EOD and LOD. According to previous studies, cognitive dysfunction (Brier et al., 2014) and affective disorder (Peng et al., 2014) can cause abnormal modularity. According to the study of Mackin et al. (2020), the LOD group showed poorer performance on measures of verbal learning and memory than the EOD group. Therefore, we speculate that the disrupted modularity of LOD may be more obvious than that of EOD. Furthermore, the effect of the longer disease course of EOD when compared with that of LOD on modularity was another aspect we needed to consider in the current study. Thus, it is hard to anticipate the modularity change between EOD and LOD, and current study was aim to explored this question.

To verify the above hypothesis, we designed this experiment. Modularity change can cause by different modular organization and/or within and between modular connectivity. Thus, first we used GC analysis to construct functional networks and test the modularity difference among groups, then applied normalized mutual information (NMI) to quantify the similarity of their modular organization, and Phi coefficient to detect the specific nodes in the modular organization. Last, within module degree z-score and participation coefficient was used to describe their within and between modular connectivity. We hope to discover the differences in the functional connections between EOD and LOD.

## Methods

### Participants

Eighty two LLD patients and 91 healthy control (HC) individuals were recruited in The Affiliated Brain Hospital of Guangzhou Medical University and a local community from September 2014 to January 2018. One of the HCs was rejected due to not undergoing a fMRI scan and removed from this study (HC#76, age: 66, gender: female, years of education: 9, HDRS: 0). All of the participants were of Han Chinese ethnicity and right-handed. Before the study, we obtained written informed consent from all of the participants. Research ethics permission for this study was given by The Affiliated Brain Hospital of Guangzhou Medical University ethics committee. The participants (LLD and HC) were the same as those used in our previous study (Mai et al., 2020).

The LLD inclusion criteria were as follows: (1) age >60 years; (2) diagnosis according to the Diagnostic and Statistical Manual of Mental Disorders, Fourth Edition (DSM-IV), criteria for major depressive disorder; (3) diagnosis was confirmed by well-trained psychiatrists from the Affiliated Brain Hospital of Guangzhou Medical University; and (4) capability to participate in a battery of neuropsychological tests. Exclusion criteria were as follows: (1) incapable of finishing the neuropsychological tests; (2) a history of major psychiatric disorders, such as bipolar affective disorder and/or schizophrenia, except depression; (3) a family history of major psychiatric disorders, such as bipolar disorder and/or schizophrenia, except depression; (4) a pre-existing primary neurological disease, such as stroke or brain tumor; and (5) a physical disease that can cause emotional problems, such as hypothyroidism or anemia.

We further divided the LLD patients into two groups, EOD and LOD. Those patients with an onset age of depression of 60 years or greater were defined as the LOD group, while the rest were defined as the EOD group.

To exclude those with mild cognitive impairment, who showed damage in functional connections greater than that expected for the elderly (Li et al., 2017), all HC were required to meet the following criteria: Mini-Mental State Examination (MMSE) scores ≥24, age>60 years and no history of depression. Other exclusion criteria were similar to those for the LLD group. Because causes of cognitive dysfunction are common in LLD, we applied the Clinical Dementia Rating (CDR)>0.5 to rule out dementia.

### Neuropsychological Tests

Participants underwent a full-scale battery of neuropsychological tests before the MRI scans. The tests were comprised of the Hamilton Rating Scale for Depression (HDRS), the Auditory Verbal Learning Test-delayed recall score, the Trail-Making Test, the Stroop Color and Word Test, the Digit Span Test, the Symbol-Digit Modality Test, the Logical Memory Test, and examination of the participants' medical records.

Cognitive function was separated into 3 domains: executive function, processing speed, and memory. The detail of each domain calculation was the same as that described in our previous article (Mai et al., 2017).

### MRI Acquisition

The experiment was carried out on a 3.0-Tesla Philips Achieva scanner (Philips, Best, The Netherlands) in the Affiliated Brain Hospital of Guangzhou Medical University. To exclude cerebral infarction, tumors, and major white matter lesions, T2-weight imaging was performed before rs-fMRI scanning. Foam padding and earplugs were used to reduce head movement and scanner noise.

Before rs-fMRI scanning, participants were told to rest and keep their eyes closed during scanning. A gradient-echo echo-planar imaging (GRE-EPI) sequence was used to perform rs-fMRI. The detailed parameters are as follows: TR = 2,000 ms, TE = 30 ms, FA = 90°, matrix = 64 × 64, slice thickness = 4 mm with inter slice gap = 0.6 mm, 33 interleaved axial slices, FOV = 220 mm × 220 mm and 240 time points.

A 3D spoiled gradient echo sequence was used to obtain high-resolution T1-weight images. The detailed parameters are: TR = 8.2 ms, TE = 3.8 ms, slice thickness = 1 mm, and FOV = 256 × 256 mm^2^ (matrix = 256 × 256 × 188).

### Data Pre-Processing

Data preprocessing for rs-fMRI was performed with GRETNA v2.0 (Wang et al., 2015), and Matlab GCA toolbox programmed by Wen et al. (2013b). The first ten time points of the functional images were removed at the beginning. Slice-timing correction was applied to the functional image for different acquisition times between slices at first and then realigned to the first time point image to correct for head movement. Images for which head movement >2 mm or head rotation >2 degrees were removed from the experiment. The realigned image was registered to MNI152 coordinates according to the 3D-T1 image by the reg_epi command in FSL5.09 (Woolrich et al., 2008). Then, spatially normalized images were resampled at 3 × 3 × 3 mm^3^ per voxel and smoothed by a Gaussian kernel with a 6 mm full width at half-maximum. The time series was extracted by Matlab and represented by a 4 dimension matrix, and then the global signal was removed by global scaling. The white matter and cerebrospinal fluid signals were removed as covariables. Finally, the fMRI signals were detrended and filtered with a temporal band-path of 0.01–0.1 Hz.

### Granger Network Construction

The brain network contains nodes and edges. In the current study, the definition of cortical areas was taken from the Human Connectome Project Multi-Modal Parcellation version 1.0 (HCP-MMP 1.0), which describes 179 cortical areas in each hemisphere. The subcortical areas referred to 14 subcorical areas according to Desikan atlas. Finally, total 372 nodes, and their MNI coordinates were determined, the detail was showed in Supplementary Table 1. An edge was defined as the GC of each node.

The GC calculation was performed by a Matlab GCA toolbox programmed by Wen et al. (2013b) to perform the analysis. The toolbox has been used in many previous studies and generated meaningful results regarding causal interactions between brain regions (Wen et al., 2012a,b, 2013a; Zhang et al., 2017). Because of the classical Granger pairwise approach may lead to ambiguous results in multivariate time settings. Wen et al. was applied the spectral density matrix to develop a multivariate Granger causality approach, the detail of the calculation was followed:

For two time series A_t_ and B_t_ can be represented by the following autoregressive models respectively:

(1) $$\begin{array}{r} {\text{A}}_{\text{t}}=\overset{\infty }{\underset{\text{j}=1}{\sum }}{\text{a}}_{1\text{j}}{\text{A}}_{\text{t}-\text{j}}\;+\;\varepsilon_{1\text{t}},\text{var}\left(\varepsilon_{1\text{t}}\right)=\Sigma_{1} \end{array}$$

(2) $$\begin{array}{r} {\text{B}}_{\text{t}}=\overset{\infty }{\underset{\text{j}=1}{\sum }}{\text{d}}_{1\text{j}}{\text{B}}_{\text{t}-\text{j}}+\eta_{1\text{t}},\text{var}\left(\eta_{1\text{t}}\right)=\Gamma_{1} \end{array}$$

They can transform into the following unrestricted bivariate AR model:

(3) $$\begin{array}{r} {\text{A}}_{\text{t}}=\overset{\infty }{\underset{\text{j}=1}{\sum }}{\text{a}}_{2\text{j}}{\text{A}}_{\text{t}-\text{j}}\;+\;\overset{\infty }{\underset{\text{j}=1}{\sum }}{\text{b}}_{2\text{j}}{\text{B}}_{\text{t}-\text{j}}+\varepsilon_{2\text{t}} \end{array}$$

(4) $$\begin{array}{r} {\text{B}}_{\text{t}}=\overset{\infty }{\underset{\text{j}=1}{\sum }}{\text{c}}_{2\text{j}}{\text{A}}_{\text{t}-\text{j}}+\overset{\infty }{\underset{\text{j}=1}{\sum }}{\text{d}}_{2\text{j}}{\text{B}}_{\text{t}-\text{j}}+\eta_{2\text{t}} \end{array}$$

The contemporaneous covariance matrix of ε_2*t*_ and η_2*t*_ are:

(5) $$\begin{array}{r} \Sigma_{2}=\;\left(\begin{array}{cc} \Sigma_{2} & \gamma_{2}\\ \gamma_{2} & \Gamma_{2} \end{array}\right) \end{array}$$

After Fourier transforming and proper ensemble average, the spectral density matrix of (1), (2) was followed:

(6) $$\begin{array}{r} \text{S}\left(\omega \right)=\text{H}\left(\omega \right)\Sigma {\text{H}}^{*}\;\left(\omega \right) \end{array}$$

*: complex conjugate and matrix transpose; H (ω): transfer function matrix.

Finally, according to Geweke (1984), the two directional measures was followed:

(7) $$\begin{array}{r} \text{G}{\text{C}}_{\text{B}\to \text{A}}\;\left(\omega \right)=\text{ln}\frac{{\text{S}}_{\text{AA}}\;\left(\omega \right)}{{\tilde{\text{H}}}_{\text{AA}}\left(\text{ω}\right)\Sigma_{2}{\tilde{\text{H}}}_{\text{AA}}^{*}\left(\omega \right)} \end{array}$$

(8) $$\begin{array}{r} \text{G}{\text{C}}_{\text{A}\to \text{B}}\;\left(\omega \right)=\text{ln}\frac{{\text{S}}_{\text{BB}}\;\left(\omega \right)}{{\tilde{\text{H}}}_{\text{BB}}\left(\omega \right)\Gamma_{2}{\tilde{\text{H}}}_{\text{BB}}^{*}{\left(\omega \right)}^{\prime }} \end{array}$$

$$\begin{array}{l} {\tilde{\text{H}}}_{\text{AA}}\left(\omega \right)={\text{H}}_{\text{AA}}\left(\omega \right)+\;\left(\gamma 2/\Sigma 2\right){\text{H}}_{\text{AB}}\;\left(\omega \right)\text{ and }\\ \;{\tilde{\text{H}}}_{\text{BB}}\left(\omega \right)={\text{H}}_{\text{BB}}\left(\omega \right)+\;\left(\gamma 2/\Gamma 2\right){\text{H}}_{\text{BA}}\;\left(\omega \right) \end{array}$$

When GC_A→B_ is the granger causal influence from node A to node B, GC_B→A_ is the granger causal influence from node B to node A. Finally, 372 × 372 directed GC connective matrices were constructed for each subject.

### Network Analysis

#### Threshold Procedure

The threshold procedure was performed before network analysis. Because of the uncertain threshold definition of the GC network, we applied sparsity to construct the series of networks under a threshold rank. The threshold procedure was performed by GRETNA v2.0 (Wang et al., 2015), the sparsity is defined as the ratio of the number of actual edges divided by the maximum possible number of edges in network, and allowing for the examination of relative network organization. The sparsity threshold was set from 0.01 to 0.5, with an interval of 0.01. Thus, a total of 50 networks according to a certain sparsity were constructed for each subject. The network properties were calculated for each threshold network. In the current study, we applied the area under the curve for different sparsity values to calculate the network properties.

#### Modularity

To describe the pattern of integration within modules and segregate between them, modularity analysis was performed. Modularity is also called Q. A larger Q indicated a greater connection than expected within the chosen modules. In the GC network, which has direction information, the calculation of the modularity was as follows (Leicht and Newman, 2008):

$$\begin{array}{l} \text{Q}=\frac{1}{\text{m}}\underset{\text{AB}}{\sum }\left[{\text{E}}_{\text{AB}}-\frac{{\text{k}}_{\text{A}}^{\text{in}}{\text{k}}_{\text{B}}^{\text{out}}}{\text{m}}\right]\delta_{{\text{c}}_{\text{A}},{\text{c}}_{\text{B}}} \end{array}$$

where m is the total weight of the edge in the network, E_AB_ is the connectivity between node A and node B; $k_{A}^{in}$ and $k_{B}^{out}\;$are the in- and out-degrees of the nodes A and B; δ_*c*_*A*__ is the Kronecker delta symbol; C_A_ is the module to which node A is assigned.

The Louvain community detection algorithm was applied in this study (Blondel et al., 2008). Considering the element of randomness that was present in this algorithm, the partition with the highest modularity was determined by 100 executions.

#### The Calculation of Normalized Mutual Information

Normalized mutual information was applied to describe the similarity between two module structures (Kuncheva and Hadjitodorov, 2004). The equation was as follows:

$$\begin{array}{l} \text{NMI}\left(\text{A},\text{B}\right)=\frac{-2\sum_{\text{i}=1}^{{\text{C}}_{\text{A}}}\sum_{\text{j}=1}^{{\text{C}}_{\text{B}}}{\text{N}}_{\text{ij}}\text{log }\left(\frac{{\text{N}}_{\text{ij}}\text{N}}{{\text{N}}_{\text{i}}{\text{N}}_{\text{j}}}\right)}{\sum_{\text{i}=1}^{{\text{C}}_{\text{A}}}{\text{N}}_{\text{i}}\text{log }\left(\frac{{\text{N}}_{\text{i}}}{\text{N}}\right)+\sum_{\text{j}=1}^{{\text{C}}_{\text{B}}}{\text{N}}_{\text{j}}\text{log }\left(\frac{{\text{N}}_{\text{j}}}{\text{N}}\right)} \end{array}$$

A and B represents the partitions of two subjects, and the number of modules in each partition are C_A_ and C_B_, respectively. N_ij_ is the number of nodes, which is the same for module i and module j and belong to subject A and subject B, respectively. N is the number of nodes, and here, *N* = 372. N_i_ is the number of nodes in module i which belong to subject A. Similarly, N_j_ is the number of nodes in module j which belong to subject B. The range of NMI was 0~1; a higher value means the partitions were more similar. In the current study, we tested whether the EOD and LOD partitions were statistically different. The within group NMI should be higher than the between group NMI. However, it is impossible to statistically analyse. According to the description of Alexander-Bloch et al. (2011), the actual within group NMI should be higher than that calculated with randomized data, while the EOD and LOD partitions were statistically different. Thus, we applied permutation testing with 10,000 samples with a custom MATLAB script to analyse the differences in the partitions between EOD and LOD. Furthermore, we applied NMI to detect the most representative subjects in the groups. By calculating the within group NMI for each pairs of subjects of the same group, we selected the higher average NMI subject as the most representative one in the 2% sparsity threshold network.

#### The Calculation of the Phi Coefficient

To detect the specific nodes in the modular assignment, we applied the Phi coefficient to describe the similarity of the modular assignment for a given node. According to the description by Alexander-Bloch et al. (2011), the other nodes, except the given node X, were binary based on whether the node was in the same module as the node X. Then, the similarity of two subjects based on the given nodes X was quantified by the Phi coefficient (−1 < ϕ < 1) (Pearson, 1900). A higher ϕ indicates a greater similarity between two subjects. Thus, if there is a difference in the node X module between EOD and LOD, the within group Phi coefficient should be higher than the between group Phi coefficient. Same as the NMI analysis, it is impossible to statistically analyse. Therefore, we a applied permutation test by randomizing the group assignment in Phi coefficient analysis. We compared the within-group average Phi coefficient in the actual data to the randomized data. The *p*-value was the number of randomized within-group average Phi coefficients that were higher than the actual within-group average Phi coefficient divided by the number of permutations (10,000 samples). Every node in the network was determined, and False Discovery Rate (FDR) correction (Benjamini and Hochberg, 1995) for multiple comparisons (*P*_fdr_ < 0.05)was performed. In the current study, we detected the specific nodes in the 2% sparsity threshold network (Alexander-Bloch et al., 2011).

#### The Calculation of the Within Module Degree Z-Score and Participation Coefficient

The within-module degree z-score quantifies how “well-connected” the given node is to other nodes in the module. Higher value indicated stronger within module connectivity of the given node. The participation coefficient quantifies how “well-distributed” the links, which are belonging to a given node, are among different modules. The value of participation coefficient is 0–1, close to 1 indicated the links are uniformly distributed among all the modules, and 0 indicated its links are within its own module.

The within-module degree z-score and participation coefficient were calculated at each node for each individual according to the identified modules. The module with <1% of the total number of nodes was excluded. The calculation of the within module degree z-score and participation coefficient was performed as described in the work of Guimerà and Nunes Amaral (2005) and performed by the Brain Connectivity Toolbox (Version 2019-03-03, http://www.nitrc.org/projects/bct).

### Statistical Analysis

The group differences in age, years of education and HRDS were determined by one-way ANOVA. The gender data was analyzed by the chi-square test.

The area under the curve of the modularity for different sparsity thresholds was calculated. The statistical analysis of all network properties was performed as the area under the curve. The area under the curve can provide a scalar that does not depend on a specific threshold selection and avoid the problem of absence definite threshold selection in network analysis. To determine the group effect of the modularity properties, the permutation test (10,000 permutations) was applied to the modularity, NMI and Phi coefficient. The significance level of permutation test was the position of the actual NMI and Phi coefficient of EOD and LOD under the distribution of NMI and Phi coefficients in randomized groups assignment. At each permutation circles, the testing subjects (belong to group_1_ or group_2_, in current study was EOD and LOD) was randomly assigned to group_x1_ and group_x2_ (x represented the number of permutation circles).

Group differences in cognitive function (memory, executive function, and processing speed), the within-module degree z-score and participation coefficient were analyzed by ANCOVA to remove the effect of age, years of education, gender and HDRS. *Post-hoc* analysis was performed using the LSD method in all of the above analyses. *P* < 0.05 was considered to be statistically significant. FDR was applied in multiple comparison corrections (*q* = 0.05).

To further determine the relationship between cognitive function, disease course and modularity, partial correlation was applied, and the effects of age, years of education, gender, and HDRS were removed. Furthermore, the relationship between the modularity and cognitive function (executive function, processing speed, and memory) was confirmed by multiple regression. Cognitive function was set as the dependent variable, and modularity was selected as the independent variable. Age, years of education, gender, and HDRS were entered in the regression equation as covariances.

The demographics, neuropsychiatric data and permutation test of the modularity, within module degree z-score and participation coefficient analyses were analyzed by IBM SPSS Statistics V22.0. The permutation test of the NMI and Phi coefficient was conducted with a custom Matlab script.

## Results

### Demographics and Neuropsychiatric Results

The demographics and neuropsychiatric data are shown in Table 1. Age and years of education were significantly different among the three groups (*P* = 0.001 & *P* = 0.002). According to the LSD *post-hoc* comparisons, EOD was lower than LOD and HC in age (*P* < 0.001 & *P* = 0.008); HC was higher in years of education than EOD and LOD (*P* = 0.001 & *P* = 0.010). No significant difference was found in the distribution of gender among the three groups (*P* = 0.197). LLD groups (EOD and LOD) were shown to have higher HDRS than HC (*P* < 0.001), while EOD had a lower HDRS than LOD (*P* = 0.009). Significant differences were found in processing speed and memory among the three groups (*P* = 0.014 & *P* = 0.018), while no significant difference was found in Executive function (*P* = 0.217). According to LSD *post-hoc* comparisons, LOD and EOD was shown to lower processing speed more than HC (*P* = 0.006 & *P* = 0.036), while EOD demonstrated a lower memory function than HC (*P* = 0.005). A negative correlation was found between HDRS and memory (*r* = −0.232, *P* = 0.002) and processing speed (*r* = −0.259, *P* = 0.002), while no correlation with HDRS was found in executive function (*P* < 0.05). The LLD groups (EOD and LOD) showed higher CDR than the HC group (*P* < 0.001), while no difference was found between EOD and LOD according to LSD *post-hoc* comparisons (*P* = 0.340).

**Table 1:** The comparison of demographic, cognitive functions and modularity between groups.

	**EOD**	**LOD**	**HC**	**F/χ^2^**	***P*-value**
	***N* = 40**	***N* = 42**	***N* = 90**		
Age	65.148 ± 4.730	70.280 ± 6.463	68.367 ± 6.868	6.908	0.001^*^
Education	8.825 ± 2.917	9.298 ± 4.525	11.000 ± 3.218	6.657	0.002^*^
Gender	8 M/32 F	12 M/30 F	32 M/58 F	3.250	0.197
HDRS	6.025 ± 5.447	8.881 ± 7.680	1.456 ± 2.152	36.625	<0.001^*^
CDR	0.450 ± 0.152	0.405 ± 0.199	0.183 ± 0.242	28.216	<0.001^*^
**Cognitive domain**					
Executive function	−0.123 ± 0.706	0.020 ± 0.531	0.041 ± 0.531	1.544	0.217
Processing speed	−0.214 ± 0.927	−0.306 ± 0.620	0.213 ± 0.610	4.392	0.014^#^
Memory	−0.313 ± 1.007	−0.280 ± 0.789	0.273 ± 0.729	4.098	0.018^#^
Q_auc_	0.067 ± 0.010	0.073 ± 0.011	0.069 ± 0.010	3.584	0.0300^#^

* *Significant according to one-way ANOVA (P < 0.05)*.

# *Significant according to one-way ANCOVA (adjusted for age, education, gender, and HDRS, P < 0.05)*.

No difference was shown between the EOD and LOD in antidepressant treatment (χ^2^ = 6.531; *P* = 0.163) and benzodiazepines (χ^2^ = 0.802; *P* = 0.370). The details are shown in Table 2.

**Table 2:** The comparison of medication information between EOD and LOD.

	**EOD**	**LOD**	**χ^2^**	***P***
	***N* = 40**	***N* = 42**		
**Antidepressants**				
No antidepressants	9	18	6.531	0.163
SNRI	7	9		
SSRI	16	11		
TCAs	2	0		
NaSSA	6	4		
**Benzodiazepines**				
With	17	22	0.802	0.370
Without	23	20		

*SNRI, serotonin noradrenaline re-uptake inhibitor; SSRI, selective serotonin reuptake inhibitors; TCAs, tricyclic antidepressants; NaSSA, noradrenergic and specific serotonergic antidepressants*.

### The Difference in Modularity

**Figure 1:**
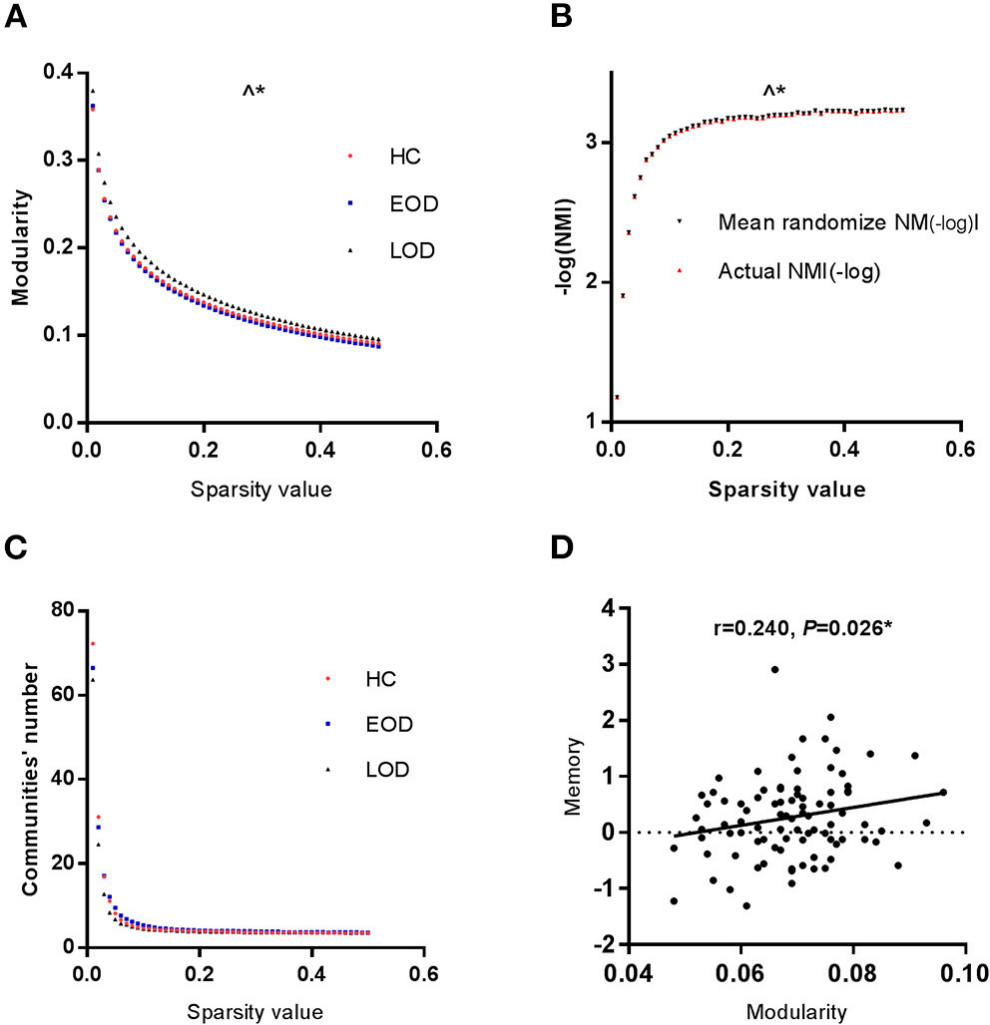
**(A)** The modularity of the GC network was significantly different among the three groups. **(B)** The NMI between EOD and LOD was significantly different. Red lines represented the-log (NMI) between EOD and LOD group. The dashed lines represented the null distribution derived from the permutation analysis. **(C)** The module number was the same in the three groups. **(D)** Modularity and memory function were positively correlated in HC group. ^∧^*P < 0.05 for the area under the curve of a range of sparsity thresholds. *P < 0.05.

The modularity of the GC network among the three groups is shown in Figure 1A. The area under the curve of the modularity in the range of the sparsity thresholds was significantly different among the three groups through permutation testing (*P*_auc_ = 0.0300, 10,000 samples, adjusted for age, education, gender, and HDRS). According to the LSD *post-hoc* comparisons, EOD was lower than LOD in modularity (*P*_auc_ = 0.0190). The partial correlation analysis demonstrated that modularity was positively correlated with memory function (*r* = 0.240, *P* = 0.026, as shown in Figure 1D) in the HC group, while no correlation was found between the disease course, cognitive function and modularity in the EOD and LOD groups. According to stepwise multiple regression after adjustment for age, year of education, gender and HDRS, modularity acted as an influence factor on memory (beta = 0.217; *P* = 0.026) in the HC group.

### The Difference in Modular Partition

According to the NMI, we analyzed the modular partition between EOD and LOD by permutation testing. The area under the curve of the within group NMI in the range of the sparsity thresholds was significantly higher than randomized effect through permutation testing (*P*_auc_ = 0.0387, 10,000 samples, The detail was showed in Figure 1B, in order to better represent the data, negative log-transformation was applied. Thus the actual NMI was larger than the randomized effect). No difference was found among the three groups in the number of modules (Figure 1C).

**Figure 2:**
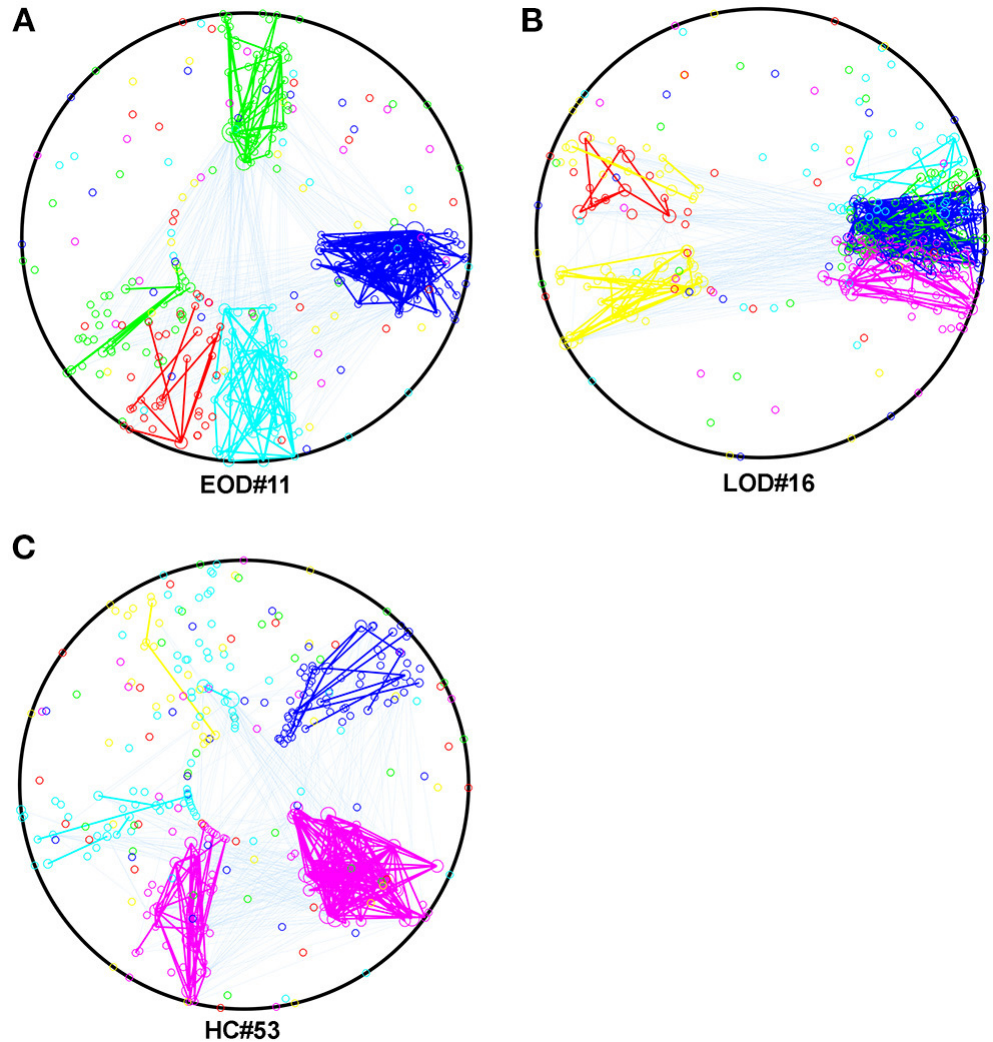
**(A–C)** The within and between module connection in EOD, LOD, and HC, respectively in 2% sparsity network, the within module connections were represented by the same color of the module, the between module connections were cyan. The color of the module was determined by the script randomly. Mapping was using The Community Detection Toolbox and Maltab home make program.

Based on the result for the NMI, we selected the most representative subjects, which had the highest within group NMI in the 2% sparsity network, and described their within and between module connections by using Community Detection Toolbox (Kehagias, 2020) and transformed them into circles by using a custom MATLAB script. The result is shown in Figures 2A–C.

### The Specific Nodes in the Modular Assignment

**Figure 3:**
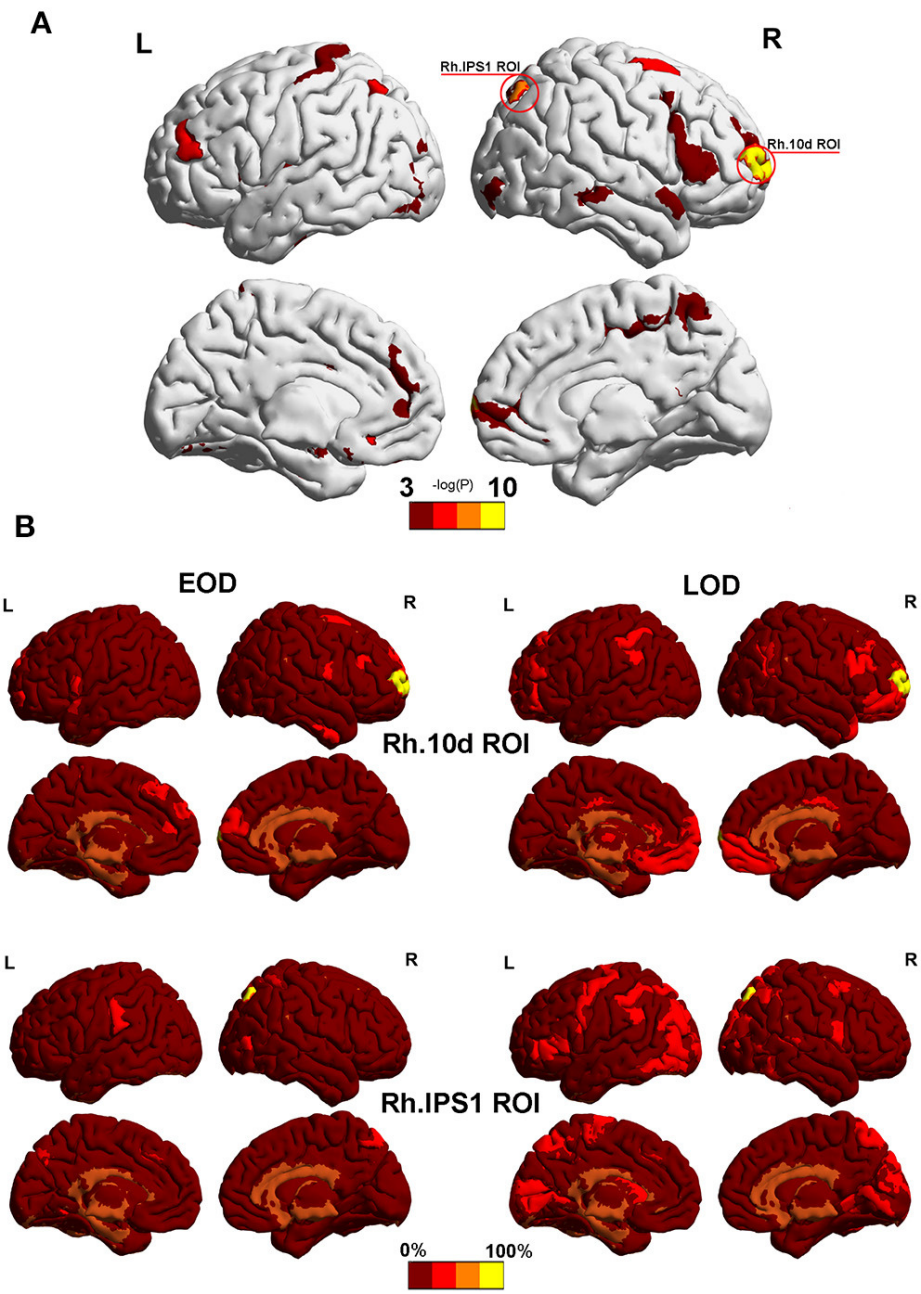
**(A)** The most significantly different partitioned nodes detected by the Phi coefficient in the 2% sparsity network. The yellow area was Rh.10d.ROI and Rh.IPS1.ROI in the HCP-MMP 1.0 atlas. **(B)** Other nodes with the same module as Rh.10d.ROI and Rh.IPS1.ROI. Color bar represents the percentage of subjects. Mapping was performed using BrainNet Viewer software (http://www.nitrc.org/projects/bnv/).

According to the Phi coefficient, we detected 2 ROI (Rh.10d.ROI and Rh.IPS1.ROI) with significant trends in different module between the EOD and LOD in the 2% sparsity network by permutation testing. The result did not pass the 5% FDR correction threshold for 372 nodes (Both *P*_fdr_ = 0.0744). Among them, Rh.10d.ROI, which belonged to the polar frontal cortex (Figure 3A), was the most differently partitioned node (*P* = 0.0002, *P*_fdr_ = 0.0744), and Rh.IPS1.ROI belonged to the intra parietal sulcus (*P* = 0.0004, *P*_fdr_ = 0.0744). The other nodes that belonged to the same module as Rh.10d.ROI and Rh.IPS1.ROI are shown in Figure 3B.

### Differences in the Within Module Degree Z-Score and Participation Coefficient

**Figure 4:**
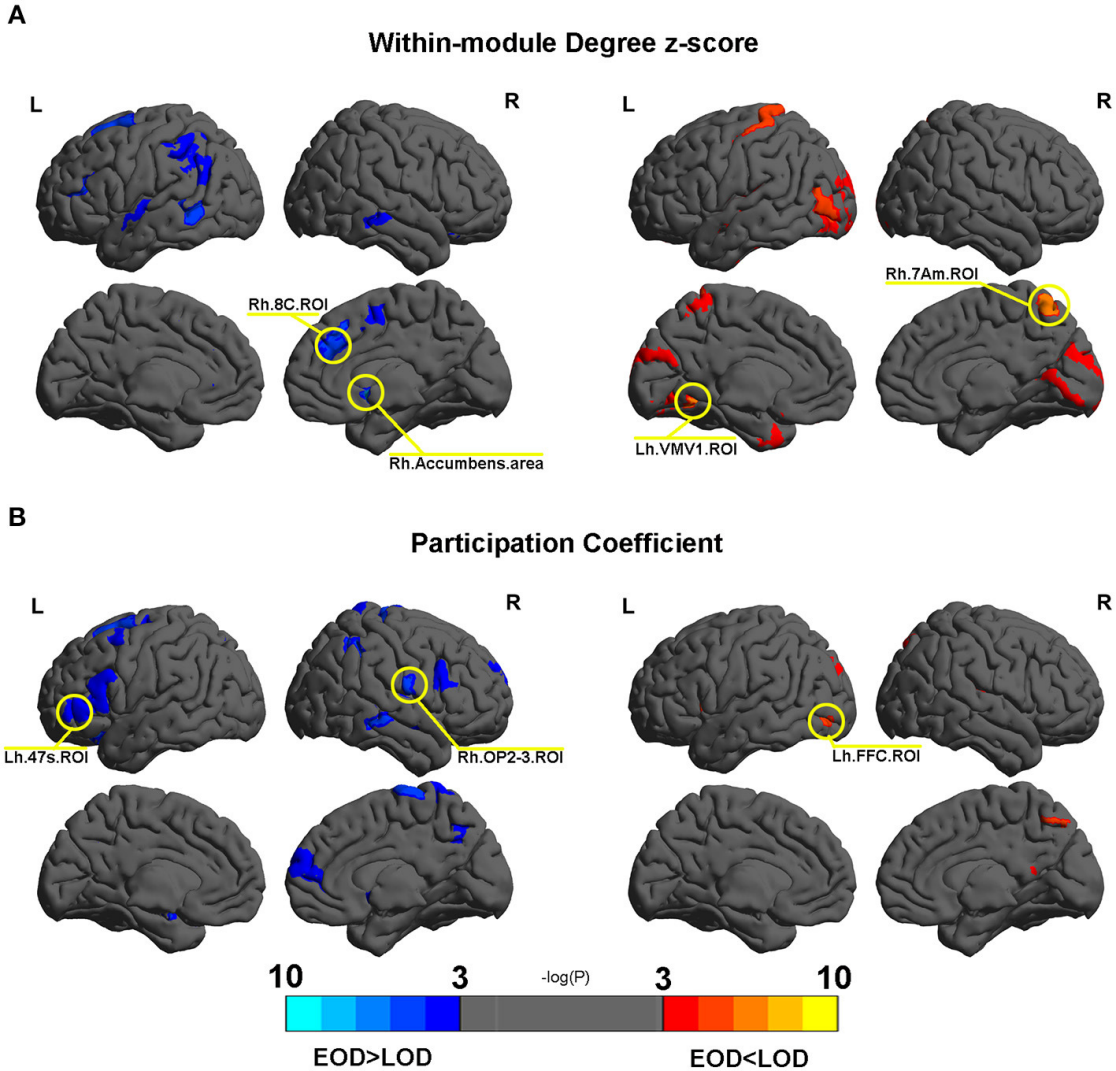
**(A,B)** The different nodes in the within module degree z-score and participation coefficient in the 2% sparsity network respectively. Mapping was performed using BrainNet Viewer software (http://www.nitrc.org/projects/bnv/).

No differences were found in the within module degree z-score and participation coefficient according to FDR correction. Several nodes were found to trend differently in terms of the within module degree z-score and participation coefficient between EOD and LOD. The details are shown in Figure 4. Among them, Rh.Accumbens.area, Lh.VMV1.ROI, Rh.7Am.ROI, and Rh.8C.ROI trended differently between EOD and LOD in terms of the within module degree z-score (*P* < 0.005). Rh.OP2-3.ROI, Lh.47s.ROI, and Lh.FFC.ROI trended significantly differently between EOD and LOD in terms of the participation coefficient (*P* < 0.005). The details of the nodes with significant trends between EOD and LOD are shown in Supplementary Tables 2, 3. Among them, Rh.Accumbens.area is the most significantly different between EOD and LOD for the within module degree z-score (*P* = 0.000494, *P*_fdr_ = 0.183748).

## Discussion

In the current study, we constructed a functional network for 82 LLD (40 EOD, and 42 LOD) and 90 HC participants according to the GC analysis and analyzed the modularity among them. First, we found that the modularity of LOD was increased compared to that of EOD in the GC network. Second, according to the NMI analysis, the partition of LOD was significantly different from that of EOD. Finally, Rh.10d.ROI and Rh.IPS1.ROI were trended to be the specific nodes associated with the modular assignment according to the Phi coefficient.

According to this study, the modularity of LOD was increased compared to that of EOD according to the GC network, and that indicated that the time-dependent modularity was different between EOD and LOD. Both EOD and LOD did not show differences compared to HC in terms of modularity, which is different from the finding of previous studies. Certain studies with various findings exist on the modularity changes in LLD (Bohr et al., 2012; Li et al., 2015). According to the findings of Li et al. (2015), the modular organization was changed in the LLD group, whereas according to Borh et al., no difference was found in the modularity between the LLD and control groups. Furthermore, none of them compared the module arrangement and special nodes that might lead to different modularities between groups. Thus, there are still far from the comprehensive understanding of modularity changes in LLD. According to us, the discrepant findings in the previous studies might because of two reasons. First, the network construction in the previous studies were based on the Pearson correlation between nodes. This ignored the time-dependency relation in the brain information process. GC can help us understand the time delay in information flow in the brain. The analysis of the modularity change in the GC network can reveal the dynamic modular change. Second, the previous studies did not divide LLD into EOD and LOD, which might have led to inconsistent findings owing to potentially different pathogenesis. Modularity is the network property used to describe the partition and integration of the network. A higher modularity indicates a higher degree of “module structure” of the network. The degree of modularity was correlated with memory function in HC. This finding did not include EOD and LOD, indicating that the module structure change caused by EOD and LOD did not reflect the memory function. According to our previous findings in rich-club analysis in white matter networks, memory function positively correlated with local connections in LLD (Mai et al., 2017). This indicates that the cognitive deficits of LLD might be caused by other mechanisms, but not by the change of functional modularity. The lower modularity of EOD in the current study might indicate that the longer disease course might have a stronger effect on modularity changes compared to cognitive deficits; however, it requires a well-designed study to confirm. Furthermore, we analyzed the weighted modularity. Thus, the increasing modularity of LOD might be caused by “lesser separation” or “greater integration” compared to those of EOD or the different module assignment between EOD and LOD. Hence, we further analyzed the difference of the module arrangement and the node-specific separation and integration properties between EOD and LOD.

The higher within group NMI coefficient compared to the randomized effect in the current study, indicated that the module arrangement was different between EOD and LOD. The NMI is within a range of 0–1. A larger NMI indicated that the partitions were similar between the two networks. According to the description of Alexander-Bloch et al. (2011), the significant higher within group NMI compared to randomized effect indicated between groups partitions were statistically different. In this study, a larger within-group average NMI compared to the randomized effect was observed, suggesting that the module arrangement was different between groups. A different module arrangement has been identified in schizophrenia compared to HC (Alexander-Bloch et al., 2011), and the change in the module arrangement might result from the disconnection caused by the disease status. Our current findings on module arrangement in EOD and LOD further support the idea that EOD and LOD might share the same clinical features but show different pathogenesis. The number of modules can affect the NMI, and our finding for the number of modules did not show any difference between groups and suggested that the difference in the NMI is caused by the module arrangement rather than the number of modules. A larger NMI implies a smaller difference between the networks in the partitions; thus, the largest average NMI indicates the most representative subjects within the groups. According to the subject selected using the largest average NMI within the group, the partitions were largely changed in LOD compared to those in EOD and HC, as shown in Figure 2. Thus, it is reasonable to speculate that the change in partitions typically occurred in LOD. However, it is still unknown whether the modularity difference between EOD and LOD was caused by the disruption of some “key” regions. Hence, we applied the Phi coefficient to determine the regions that were different in the partitions between groups.

We specified the two regions that showed a trend of discrepancy in the partitions between EOD and LOD by an analysis of the Phi coefficient. Rh.10d.ROI belonged to the polar frontal cortex. Recent evidence indicate that the frontal cortex not only affects motor ability but also sensory integration, behavioral strategizing, working memory, and decision-making (Ebbesen et al., 2018). Rh.IPS1.ROI belonged to the dorsal stream visual cortex, and it was connected with anterior areas along the border between the visual and cognitively relevant regions. These two regions were associated with cognitive function to some extent. However, no difference was found between the EOD and LOD in the cognitive domain. In addition to the identification of partitions, it was indicated that the cognitive function changed less than expected as the partitions altered. It is still unknown whether the unique modules including Rh.10d.ROI and Rh.IPS1.ROI might contribute to further cognitive discrepancies between EOD and LOD in the future, such as dementia owing to LOD; there is a need for further study to analyse the effects of these two regions on cognitive functions in LLD.

The change in the weight modularity might not only result from different partitions, but also differences within and/or between module connections. This can be tested with the within module degree z-score and participation coefficient. However, no difference was found in these properties in the current study. In addition to the findings of the NMI analysis in the current study, it was indicated that the different modularity values between EOD and LOD were typically caused by the differences in the modularity arrangement. However, some regions showed different trends between EOD and LOD. Among them, the Rh.Accumbens.area appeared to be the most likely to be different in terms of the within module degree z-score between EOD and LOD. The accumbens is involved in brain reward circles (Russo and Nestler, 2013), and reward deficits are one of the important characteristics of depression. The difference of the within module degree z-score for Rh.Accumbens.area might indicate different reward guiding behaviors. Our previous finding showed abnormal altruistic behavior in LLD participants (Wu et al., 2020). However, as the result is far from the standard of FDR correction, we should consider this finding carefully.

## Limitation

There are some limitations of the current study. First, the participants were not medication-naive subjects. However, the medication record did not differ between the EOD and LOD. Thus, we believe that the effect of medications had limited effects on the results of the current study. Second, we did not repeat our experiment at a different time resolution. However, the relationship between time resolution and Granger function network has not been fully investigated, and our future study will try to explore whether this effect exists. Third, the current study on Granger causality analysis was based on linear model, which has been widely used. However, it is widely assumed that functional connectivity is highly non-linear (Marinazzo et al., 2011). Furthermore, Granger causality analysis would affect by the confounding effect of hemodynamic response function (Wu et al., 2013), which is also considered as an example of non-linear function of physiological parameters. Thus, the non-linear Granger causality analysis, such as Kernel Granger causality should be included in our further study, and the effect of hemodynamic response function should also be considered. Fourth, the HDRS was different between EOD and LOD, and some studies have found an association between HDRS and functional connection (Wang et al., 2019; Weng et al., 2019); hence, we applied HDRS as a covariate in our analysis. No correlation was found between modularity and HDRS (shown in Supplementary Figure 1). Thus, we believe that the differences in HDRS have limited influence on our findings.

## Conclusion

In this study, we found that the functional modularity was different between EOD and LOD. Our findings in terms of NMI for the partition, the within module degree z-score and participation coefficient confirmed that the modularity change between EOD and LOD was mainly caused by the change in the partition but not within and between modular connections. These findings support the hypothesis that different pathological changes might exist in EOD and LOD.

## Data Availability Statement

The raw data supporting the conclusions of this article will be made available by the authors, without undue reservation.

## Ethics Statement

The studies involving human participants were reviewed and approved by The Affiliated Brain Hospital of Guangzhou Medical University ethics committee. The patients/participants provided their written informed consent to participate in this study.

## Author Contributions

NM was responsible for the data analysis. NM and YW were writing the drafts of manuscript. YN supervised experiment design and the drafts of the manuscript. NM, YW, XZ, BC, MZ, and QP were involved in data collection. All authors approved the final version of the manuscript.

## Conflict of Interest

The authors declare that the research was conducted in the absence of any commercial or financial relationships that could be construed as a potential conflict of interest.
