# Supplementary material for: Different Modular Organization Between Early Onset and Late Onset Depression: A Study Base on Granger Causality Analysis
Source: Front Aging Neurosci. 2021 Feb 9;13:625175. doi: 10.3389/fnagi.2021.625175 (PMC7900556; doi:10.3389/fnagi.2021.625175)
Supplement: Supplementary file 1 [file Data_Sheet_1.pdf]

Supplemental Table 1. List of total 372 nodes, and their MNI coordinates

ROI	MNI coordinates X	Y	Z	ROI	MNI coordinates X	Y	Z
Left-Accumbens-area	-9	13	-7	Right-Accumbens-area	10	14	-7
Left-Amygdala	-23	-2	-19	Right-Amygdala	25	-1	-19
Left-Caudate	-14	8	11	Right-Caudate	14	10	10
Left-Hippocampus	-26	-22	-12	Right-Hippocampus	27	-19	-12
Left-Pallidum	-20	-2	-2	Right-Pallidum	20	-1	-1
Left-Putamen	-26	2	0	Right-Putamen	26	4	0
Left-Thalamus-Proper	-13	-17	6	Right-Thalamus-Proper	10	-16	6
Lh.L_10d_ROI	-10	65	11	Rh.R_10d_ROI	11	66	6
Lh.L_10pp_ROI	-12	62	-13	Rh.R_10pp_ROI	13	61	-13
Lh.L_10r_ROI	-6	52	-7	Rh.R_10r_ROI	9	48	-8
Lh.L_10v_ROI	-4	53	-15	Rh.R_10v_ROI	6	51	-14
Lh.L_11l_ROI	-25	47	-14	Rh.R_11l_ROI	26	44	-16
Lh.L_13l_ROI	-22	30	-19	Rh.R_13l_ROI	22	28	-20
Lh.L_1_ROI	-44	-26	57	Rh.R_1_ROI	47	-26	54
Lh.L_23c_ROI	-11	-28	47	Rh.R_23c_ROI	9	-31	48
Lh.L_23d_ROI	-3	-21	41	Rh.R_23d_ROI	4	-22	42
Lh.L_24dd_ROI	-6	-18	54	Rh.R_24dd_ROI	7	-18	56
Lh.L_24dv_ROI	-9	-4	48	Rh.R_24dv_ROI	8	-6	49
Lh.L_25_ROI	-5	23	-11	Rh.R_25_ROI	4	23	-12
Lh.L_2_ROI	-38	-33	51	Rh.R_2_ROI	36	-33	50
Lh.L_31a_ROI	-5	-39	48	Rh.R_31a_ROI	5	-40	47
Lh.L_31pd_ROI	-9	-50	39	Rh.R_31pd_ROI	9	-52	37
Lh.L_31pv_ROI	-8	-46	36	Rh.R_31pv_ROI	7	-44	37
Lh.L_33pr_ROI	-4	14	28	Rh.R_33pr_ROI	4	11	28
Lh.L_3a_ROI	-30	-20	52	Rh.R_3a_ROI	36	-20	46
Lh.L_3b_ROI	-36	-23	56	Rh.R_3b_ROI	39	-23	53
Lh.L_43_ROI	-56	-2	7	Rh.R_43_ROI	60	3	10
Lh.L_44_ROI	-56	13	21	Rh.R_44_ROI	54	17	22
Lh.L_45_ROI	-51	26	7	Rh.R_45_ROI	55	26	6
Lh.L_46_ROI	-36	43	28	Rh.R_46_ROI	35	45	26
Lh.L_47l_ROI	-47	27	-6	Rh.R_47l_ROI	49	26	-9
Lh.L_47m_ROI	-37	29	-12	Rh.R_47m_ROI	35	32	-17
Lh.L_47s_ROI	-32	23	-18	Rh.R_47s_ROI	32	22	-19
Lh.L_4_ROI	-28	-16	57	Rh.R_4_ROI	28	-19	59
Lh.L_52_ROI	-40	-18	0	Rh.R_52_ROI	38	-19	4
Lh.L_55b_ROI	-48	1	47	Rh.R_55b_ROI	50	-2	49
Lh.L_5L_ROI	-8	-45	70	Rh.R_5L_ROI	11	-46	77
Lh.L_5m_ROI	-6	-35	72	Rh.R_5m_ROI	4	-39	66
Lh.L_5mv_ROI	-13	-36	52	Rh.R_5mv_ROI	11	-38	54
Lh.L_6a_ROI	-24	0	54	Rh.R_6a_ROI	29	-1	54
Lh.L_6d_ROI	-36	-16	66	Rh.R_6d_ROI	40	-15	62

Lh.L_6ma_ROI	-21	7	65	Rh.R_6ma_ROI	20	7	65
Lh.L_6mp_ROI	-18	-10	71	Rh.R_6mp_ROI	21	-13	68
Lh.L_6r_ROI	-53	5	17	Rh.R_6r_ROI	51	8	20
Lh.L_6v_ROI	-57	6	32	Rh.R_6v_ROI	57	5	35
Lh.L_7AL_ROI	-16	-55	68	Rh.R_7AL_ROI	17	-56	73
Lh.L_7Am_ROI	-8	-59	58	Rh.R_7Am_ROI	5	-58	63
Lh.L_7m_ROI	-4	-62	37	Rh.R_7m_ROI	5	-64	36
Lh.L_7PC_ROI	-31	-55	62	Rh.R_7PC_ROI	33	-52	64
Lh.L_7PL_ROI	-12	-74	56	Rh.R_7PL_ROI	9	-71	60
Lh.L_7Pm_ROI	-4	-69	51	Rh.R_7Pm_ROI	8	-68	50
Lh.L_8Ad_ROI	-22	29	42	Rh.R_8Ad_ROI	23	30	38
Lh.L_8Av_ROI	-34	22	52	Rh.R_8Av_ROI	40	21	45
Lh.L_8BL_ROI	-11	37	51	Rh.R_8BL_ROI	15	42	48
Lh.L_8BM_ROI	-3	30	46	Rh.R_8BM_ROI	7	30	47
Lh.L_8C_ROI	-43	18	40	Rh.R_8C_ROI	37	22	35
Lh.L_9-46d_ROI	-28	46	20	Rh.R_9-46d_ROI	30	50	17
Lh.L_9a_ROI	-19	57	27	Rh.R_9a_ROI	20	63	17
Lh.L_9m_ROI	-5	54	25	Rh.R_9m_ROI	8	53	20
Lh.L_9p_ROI	-20	45	41	Rh.R_9p_ROI	19	53	30
Lh.L_a10p_ROI	-28	57	-6	Rh.R_a10p_ROI	27	59	-10
Lh.L_A1_ROI	-43	-20	10	Rh.R_A1_ROI	44	-20	11
Lh.L_a24pr_ROI	-6	20	31	Rh.R_a24pr_ROI	5	15	33
Lh.L_a24_ROI	-6	44	0	Rh.R_a24_ROI	7	38	0
Lh.L_a32pr_ROI	-10	32	30	Rh.R_a32pr_ROI	9	25	31
Lh.L_a47r_ROI	-40	44	-12	Rh.R_a47r_ROI	41	47	-11
Lh.L_A4_ROI	-62	-19	8	Rh.R_A4_ROI	65	-15	4
Lh.L_A5_ROI	-60	-12	-1	Rh.R_A5_ROI	62	-12	-3
Lh.L_a9-46v_ROI	-41	51	5	Rh.R_a9-46v_ROI	43	50	5
Lh.L_AAIC_ROI	-32	15	-11	Rh.R_AAIC_ROI	34	17	-10
Lh.L_AIP_ROI	-38	-38	46	Rh.R_AIP_ROI	36	-40	47
Lh.L_AVI_ROI	-31	25	-1	Rh.R_AVI_ROI	34	26	-3
Lh.L_d23ab_ROI	-4	-41	32	Rh.R_d23ab_ROI	4	-37	36
Lh.L_d32_ROI	-7	42	27	Rh.R_d32_ROI	9	37	25
Lh.L_DVT_ROI	-19	-69	32	Rh.R_DVT_ROI	21	-65	34
Lh.L_EC_ROI	-20	-9	-27	Rh.R_EC_ROI	23	-9	-24
Lh.L_FEF_ROI	-38	-2	56	Rh.R_FEF_ROI	44	-7	51
Lh.L_FFC_ROI	-38	-62	-15	Rh.R_FFC_ROI	39	-56	-18
Lh.L_FOP1_ROI	-51	5	5	Rh.R_FOP1_ROI	51	7	5
Lh.L_FOP2_ROI	-43	-2	13	Rh.R_FOP2_ROI	42	-1	13
Lh.L_FOP3_ROI	-35	5	14	Rh.R_FOP3_ROI	36	8	12
Lh.L_FOP4_ROI	-42	14	10	Rh.R_FOP4_ROI	41	15	8
Lh.L_FOP5_ROI	-35	26	8	Rh.R_FOP5_ROI	39	26	7
Lh.L_FST_ROI	-45	-65	0	Rh.R_FST_ROI	45	-68	-4
Lh.L_i6-8_ROI	-29	11	55	Rh.R_i6-8_ROI	33	12	55

Lh.L_IFJa_ROI	-41	12	27	Rh.R_IFJa_ROI	38	21	26
Lh.L_IFJp_ROI	-40	5	30	Rh.R_IFJp_ROI	34	7	30
Lh.L_IFSa_ROI	-46	32	12	Rh.R_IFSa_ROI	50	36	8
Lh.L_IFSp_ROI	-47	26	25	Rh.R_IFSp_ROI	48	30	24
Lh.L_Ig_ROI	-35	-13	15	Rh.R_Ig_ROI	36	-11	15
Lh.L_IP0_ROI	-29	-78	30	Rh.R_IP0_ROI	33	-71	33
Lh.L_IP1_ROI	-31	-66	39	Rh.R_IP1_ROI	37	-63	50
Lh.L_IP2_ROI	-40	-46	45	Rh.R_IP2_ROI	42	-45	46
Lh.L_IPS1_ROI	-25	-72	38	Rh.R_IPS1_ROI	28	-68	44
Lh.L_LBelt_ROI	-45	-23	8	Rh.R_LBelt_ROI	48	-22	8
Lh.L_LIPd_ROI	-30	-53	39	Rh.R_LIPd_ROI	30	-55	49
Lh.L_LIPv_ROI	-32	-61	50	Rh.R_LIPv_ROI	26	-58	56
Lh.L_LO1_ROI	-39	-82	9	Rh.R_LO1_ROI	39	-81	6
Lh.L_LO2_ROI	-39	-88	-2	Rh.R_LO2_ROI	45	-79	-3
Lh.L_LO3_ROI	-42	-78	18	Rh.R_LO3_ROI	43	-80	14
Lh.L_MBelt_ROI	-46	-14	2	Rh.R_MBelt_ROI	46	-14	6
Lh.L_MIP_ROI	-21	-64	42	Rh.R_MIP_ROI	24	-67	58
Lh.L_MI_ROI	-36	11	4	Rh.R_MI_ROI	39	11	3
Lh.L_MST_ROI	-43	-66	8	Rh.R_MST_ROI	45	-69	8
Lh.L_MT_ROI	-44	-73	13	Rh.R_MT_ROI	45	-71	12
Lh.L_OFC_ROI	-10	36	-21	Rh.R_OFC_ROI	10	36	-22
Lh.L_OP1_ROI	-45	-19	17	Rh.R_OP1_ROI	43	-19	18
Lh.L_OP2-3_ROI	-40	-13	17	Rh.R_OP2-3_ROI	40	-11	18
Lh.L_OP4_ROI	-60	-9	15	Rh.R_OP4_ROI	57	-9	14
Lh.L_p10p_ROI	-24	61	5	Rh.R_p10p_ROI	27	58	3
Lh.L_p24pr_ROI	-4	-1	40	Rh.R_p24pr_ROI	5	-5	40
Lh.L_p24_ROI	-4	40	19	Rh.R_p24_ROI	6	34	17
Lh.L_p32pr_ROI	-6	14	41	Rh.R_p32pr_ROI	8	11	42
Lh.L_p32_ROI	-13	50	0	Rh.R_p32_ROI	10	46	-1
Lh.L_p47r_ROI	-44	43	2	Rh.R_p47r_ROI	46	42	-7
Lh.L_p9-46v_ROI	-43	35	29	Rh.R_p9-46v_ROI	41	40	25
Lh.L_PBelt_ROI	-52	-22	9	Rh.R_PBelt_ROI	58	-18	8
Lh.L_PCV_ROI	-8	-51	50	Rh.R_PCV_ROI	6	-53	50
Lh.L_PeEc_ROI	-29	-7	-34	Rh.R_PeEc_ROI	30	-7	-32
Lh.L_PEF_ROI	-44	8	42	Rh.R_PEF_ROI	44	-1	41
Lh.L_PFcm_ROI	-48	-29	18	Rh.R_PFcm_ROI	48	-30	21
Lh.L_PFm_ROI	-49	-47	45	Rh.R_PFm_ROI	52	-49	39
Lh.L_PFop_ROI	-59	-19	20	Rh.R_PFop_ROI	63	-21	22
Lh.L_PF_ROI	-58	-29	34	Rh.R_PF_ROI	59	-32	37
Lh.L_PFt_ROI	-49	-21	33	Rh.R_PFt_ROI	52	-23	35
Lh.L_PGi_ROI	-49	-57	26	Rh.R_PGi_ROI	45	-58	24
Lh.L_PGp_ROI	-36	-81	28	Rh.R_PGp_ROI	40	-77	27
Lh.L_PGs_ROI	-43	-69	38	Rh.R_PGs_ROI	44	-66	39
Lh.L_PHA1_ROI	-19	-33	-12	Rh.R_PHA1_ROI	20	-32	-13

Lh.L_PHA2_ROI	-27	-35	-11	Rh.R_PHA2_ROI	30	-31	-14
Lh.L_PHA3_ROI	-29	-36	-16	Rh.R_PHA3_ROI	32	-34	-15
Lh.L_PH_ROI	-44	-71	-7	Rh.R_PH_ROI	47	-64	-11
Lh.L_PHT_ROI	-57	-56	-4	Rh.R_PHT_ROI	57	-51	-2
Lh.L_PI_ROI	-44	2	-14	Rh.R_PI_ROI	47	-1	-11
Lh.L_Pir_ROI	-30	8	-18	Rh.R_Pir_ROI	34	9	-18
Lh.L_PIT_ROI	-30	-88	-7	Rh.R_PIT_ROI	39	-84	-12
Lh.L_pOFC_ROI	-14	18	-18	Rh.R_pOFC_ROI	15	18	-17
Lh.L_PoI1_ROI	-39	-9	-3	Rh.R_PoI1_ROI	39	-9	-3
Lh.L_PoI2_ROI	-40	-2	1	Rh.R_PoI2_ROI	42	-2	1
Lh.L_POS1_ROI	-12	-56	14	Rh.R_POS1_ROI	11	-54	17
Lh.L_POS2_ROI	-9	-71	38	Rh.R_POS2_ROI	13	-71	39
Lh.L_PreS_ROI	-20	-28	-9	Rh.R_PreS_ROI	19	-27	-9
Lh.L_ProS_ROI	-22	-54	6	Rh.R_ProS_ROI	20	-45	6
Lh.L_PSL_ROI	-59	-44	26	Rh.R_PSL_ROI	59	-41	25
Lh.L_RI_ROI	-40	-32	19	Rh.R_RI_ROI	42	-31	18
Lh.L_RSC_ROI	-8	-36	21	Rh.R_RSC_ROI	6	-33	24
Lh.L_s32_ROI	-6	35	-13	Rh.R_s32_ROI	6	33	-12
Lh.L_s6-8_ROI	-17	29	56	Rh.R_s6-8_ROI	22	23	54
Lh.L_SCEF_ROI	-6	3	61	Rh.R_SCEF_ROI	6	2	63
Lh.L_SFL_ROI	-7	18	65	Rh.R_SFL_ROI	9	17	66
Lh.L_STGa_ROI	-51	16	-17	Rh.R_STGa_ROI	53	17	-18
Lh.L_STSda_ROI	-52	-4	-11	Rh.R_STSda_ROI	53	-2	-13
Lh.L_STSdp_ROI	-52	-31	3	Rh.R_STSdp_ROI	52	-28	-1
Lh.L_STSva_ROI	-50	-6	-17	Rh.R_STSva_ROI	56	-10	-15
Lh.L_STSvp_ROI	-58	-30	-2	Rh.R_STSvp_ROI	60	-33	-6
Lh.L_STV_ROI	-58	-48	14	Rh.R_STV_ROI	57	-43	14
Lh.L_TA2_ROI	-51	5	-7	Rh.R_TA2_ROI	54	4	-5
Lh.L_TE1a_ROI	-58	-5	-23	Rh.R_TE1a_ROI	61	-2	-23
Lh.L_TE1m_ROI	-62	-22	-10	Rh.R_TE1m_ROI	62	-28	-16
Lh.L_TE1p_ROI	-64	-41	-11	Rh.R_TE1p_ROI	60	-46	-14
Lh.L_TE2a_ROI	-53	-24	-25	Rh.R_TE2a_ROI	55	-15	-29
Lh.L_TE2p_ROI	-46	-47	-18	Rh.R_TE2p_ROI	49	-41	-20
Lh.L_TF_ROI	-40	-21	-26	Rh.R_TF_ROI	42	-23	-26
Lh.L_TGd_ROI	-37	16	-31	Rh.R_TGd_ROI	39	16	-34
Lh.L_TGv_ROI	-38	4	-39	Rh.R_TGv_ROI	35	1	-42
Lh.L_TPOJ1_ROI	-54	-46	6	Rh.R_TPOJ1_ROI	52	-41	8
Lh.L_TPOJ2_ROI	-52	-62	9	Rh.R_TPOJ2_ROI	55	-60	4
Lh.L_TPOJ3_ROI	-42	-68	19	Rh.R_TPOJ3_ROI	52	-63	14
Lh.L_V1_ROI	-8	-83	3	Rh.R_V1_ROI	14	-77	4
Lh.L_v23ab_ROI	-6	-53	23	Rh.R_v23ab_ROI	6	-49	24
Lh.L_V2_ROI	-10	-80	7	Rh.R_V2_ROI	13	-76	7
Lh.L_V3A_ROI	-17	-89	33	Rh.R_V3A_ROI	21	-86	34
Lh.L_V3B_ROI	-26	-80	24	Rh.R_V3B_ROI	28	-75	22

Lh.L_V3CD_ROI	-31	-86	17	Rh.R_V3CD_ROI	33	-85	19
Lh.L_V3_ROI	-14	-86	8	Rh.R_V3_ROI	20	-84	9
Lh.L_V4_ROI	-26	-87	4	Rh.R_V4_ROI	30	-85	2
Lh.L_V4t_ROI	-46	-77	2	Rh.R_V4t_ROI	47	-75	1
Lh.L_V6A_ROI	-19	-80	44	Rh.R_V6A_ROI	29	-80	46
Lh.L_V6_ROI	-11	-78	34	Rh.R_V6_ROI	18	-72	32
Lh.L_V7_ROI	-21	-79	35	Rh.R_V7_ROI	28	-78	34
Lh.L_V8_ROI	-26	-77	-13	Rh.R_V8_ROI	26	-77	-12
Lh.L_VIP_ROI	-23	-66	56	Rh.R_VIP_ROI	15	-60	63
Lh.L_VMV1_ROI	-18	-51	-4	Rh.R_VMV1_ROI	17	-49	-4
Lh.L_VMV2_ROI	-28	-51	-5	Rh.R_VMV2_ROI	28	-51	-6
Lh.L_VMV3_ROI	-29	-62	-9	Rh.R_VMV3_ROI	27	-60	-8
Lh.L_VVC_ROI	-29	-52	-15	Rh.R_VVC_ROI	27	-47	-16

Supplemental Table 2 The ROI with different participation coefficient between EOD and LOD

	F	*P*
Rh.OP2.3.ROI	10. 89336	0. 001471
Lh.47s.ROI	10. 25897	0. 001988
Lh.FFC.ROI	8. 597889	0. 004445
Rh.IP2.ROI	7. 248783	0. 008724
Rh.7Pm.ROI	7. 039937	0. 009702
Rh.4.ROI	6. 717525	0. 011443
Rh.STSdp.ROI	6. 046562	0. 016208
Lh.Amygdala	6. 042305	0. 016244
Lh.6a.ROI	5. 955372	0. 017002
Lh.MI.ROI	5. 892055	0. 017577
Rh.5L.ROI	5. 696017	0. 019492
Lh.p47r.ROI	5. 633629	0. 020146
Rh.OP4.ROI	5. 053207	0. 02748
Rh.IFJa.ROI	4. 811764	0. 031324
Rh.DVT.ROI	4. 734196	0. 032677
Lh.6d.ROI	4. 701463	0. 033266
Lh.45.ROI	4. 543965	0. 036267
Rh.Accumbens.area	4. 370714	0. 039905
Rh.9m.ROI	4. 363168	0. 040072
Rh.IFSp.ROI	4. 325778	0. 040911
Lh.PBelt.ROI	4. 310178	0. 041267
Rh.7m.ROI	4. 163195	0. 044785

Supplemental Table 3. The ROI with different within module degree z-score between EOD and LOD

	F	P
Rh.Accumbens.area	13.25423	0.000494
Lh.VMV1.ROI	10.37802	0.001878
Rh.7Am.ROI	10.19616	0.002048
Rh.8C.ROI	8.554824	0.00454
Lh.PIT.ROI	8.314145	0.005114
Lh.V4t.ROI	7.444993	0.007899
Lh.PHT.ROI	7.080611	0.009502
Rh.a32pr.ROI	7.025823	0.009772
Lh.IFSp.ROI	7.012233	0.00984
Lh.1.ROI	6.501074	0.012795
Lh.LO2.ROI	6.35731	0.013784
Lh.6a.ROI	6.269175	0.01443
Lh.IFSa.ROI	6.194229	0.015004
Lh.FFC.ROI	5.800807	0.018442
Rh.PIT.ROI	5.389575	0.022939
Lh.Pallidum	5.18984	0.02553
Lh.RI.ROI	5.156159	0.025997
Rh.STSdp.ROI	5.147003	0.026125
Lh.V4.ROI	5.040348	0.027671
Lh.5L.ROI	4.822085	0.031148
Rh.IP0.ROI	4.623312	0.034721
Rh.a24pr.ROI	4.407938	0.039092
Rh.V2.ROI	4.323035	0.040973
Lh.PGi.ROI	4.209164	0.043651
Lh.LO3.ROI	4.150074	0.045115
Lh.FOP3.ROI	4.115927	0.045984
Rh.24dv.ROI	4.015546	0.048647
Rh.47s.ROI	4.009236	0.048819

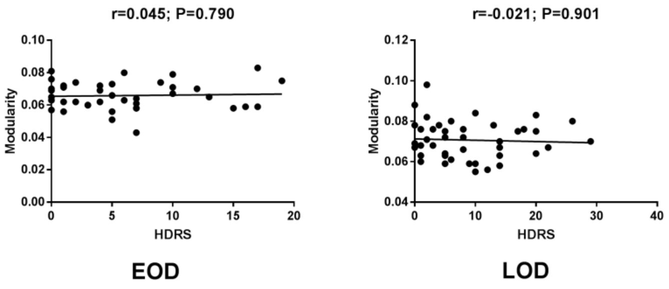

Supplemental Figure 1. The correlation between modularity and HDRS in LLD subjects (EOD and LOD) from (adjusted gender, years of education and age).
